# Aluminiums for thixoforging and preparatory conditions and parameters necessary before forming: A review

**DOI:** 10.1016/j.heliyon.2023.e22332

**Published:** 2023-11-17

**Authors:** Adriana Neag, Eric Becker

**Affiliations:** a Technical University, Materials Science and Engineering Department, 103–105 Bwd. Muncii, 400641, Cluj-Napoca, Romania; b Laboratoire de Conception, Fabrication et Commandes – Arts et Métiers, ENSAM - 4, rue Augustin Fresnel, F57078, Metz, France

**Keywords:** Thixoforging, Aluminium, Semi-solid, DSC, Process parameters

## Abstract

The thixoforging of aluminium alloys has received in recent years an extensive interest for the manufacture of parts because of its advantages. To choose an aluminium alloy as raw material for a thixoforming process, it is important to know, the product characteristics, respectively the expected mechanical properties, and secondly, the semi-solid interval and the temperature of fusion. Various wrought and foundry aluminium alloys are thoroughly summarized in this review paper, together with a description of the evaluation methods for the heating temperature sensitivity. Furthermore, for thixoforging industrial applications, the heating strategy is very important, and the reheating regime should be systematically analysed. The advantages offered by thixoforging of aluminium alloys are associated to the feedstock material microstructure that results after reheating to the semi-solid range, which is key to understand the rheological behaviour and the final mechanical properties of the thixoformed parts. In the future, a process model needs to be developed to integrate microstructure conditioning during reheating of the material to the semi-solid state.

## 1 Introduction

The major *manufacturing industries* are in continuous development, both technologically and economically, but also environmentally. The development of more efficient production processes for the near net shape processing of products made from materials that could be shaped only with difficulty by other techniques is an essential issue for commercial success. This challenge is constantly explored by several authors.

The thixoforming is a competitive technology capable of fabricating near-net-shape parts that require semi-solid metal forming. This new kind of process, introduced by Merton C. Flemings at MIT in the 1970s, allows good flow capacity and the advantage of a low resistance to deformation [1]. The behaviour of semi-solids used for thixoforming implies a decrease in alloy viscosity when subjected to shear stress and will thicken again if allowed to stand; their viscosity depends on time and shear rate [2,3]. Thixoforming consists of processing alloys in the semi-solid state, preferably with a spheroidal microstructure to further improve the forming result. The rheological properties of the non-dendritic semi-solid microstructure can easily be achieved by cooling the alloy from the liquid state or by reheating the solid alloys in the semi-solid temperature range [4]. Until today, several methods that allow the production of this target microstructure were presented by the researchers. Trying to improve the industry response, nowadays different researchers publish the results of the thixoforming processes, showing both advantages but also some limitations of the methods.

Maintaining some of the advantages of hot forming, the thixoforming process has specific advantages resulting from the materials’ rheological properties. To achieve these benefits, it is necessary to know the specific particularities related to the feedstock manufacturing, reheating parameters, and forming processes. The major disadvantage of the thixoforming processes is the frequent treatment of the raw material until forming to improve the flow and part characteristics.

It is well known that the flow capacity of a material during the forming step is influenced by its microstructure. The semi-solid route requires preferably an alloy with a non-dendritic microstructure, i.e. a suspension of solid, nearly globular with primary particles surrounded by liquid matrix necessary to provide a thixotropic flow. Feedstock quality, respectively the size and shape of the globules and the quantity of the solid phase, must be controlled, in order to benefit from an appropriate viscosity of the semi-solid material. The characteristics of the non-dendritic microstructure play a key role in the semi-solid materials processing [5].

On the other hand, the forming parameter: die temperature, ram speed and pressure have an important effect on forming process results and are the subject of several interesting papers.

In the literature, many articles are devoted to the shaping of aluminium alloys by thixoforming; evaluation of the solid or liquid fraction of an alloy grade in the semi-solid state, process factors that may affect it, preparation of the material to improve shaping, etc. However, the search for exhaustive articles summarizing the different grades used, their specific preparation, choices elements for improving the process or its understanding, has been unsuccessful. The literature on the thixoforging of aluminium presents articles that compare research work with a limited:

• number of grades, or only one, and they are often close together,
• parameters for the production process of an aluminium alloy in the semi-solid state,
• criteria for the choice of metal according to the expected application or a small number of thixoformability criteria.

The objectives of the article are to produce a synthesis of data from scientific articles on the use of aluminium alloys that can be shaped via thixoforming processing routes and the elements needed to select and prepare them. It reviews:

• the relevant thixoformed aluminium alloys necessary to know before forming at the semi-solid state,
• the criteria necessary to identify the raw material, suitable for thixoforming,
• the semi-solid preparation methods reported in the literature,
• the microstructure/morphology characterization of semi-solid raw aluminium.

The paper provides also information on the typical morphology of the remaining solid phases, which influence the rheology of the semi-solid material. The mechanical properties of the thixoformed parts or the forming step are not presented and analysed here.

### 1.1 Relevant aluminium alloys identification

Over the past few years, different types of aluminium alloys have been studied in terms of their behaviour during thixoforming. The alloys that deserve attention belong to both the wrought and foundry aluminium alloys families.

**Fig. 1:**
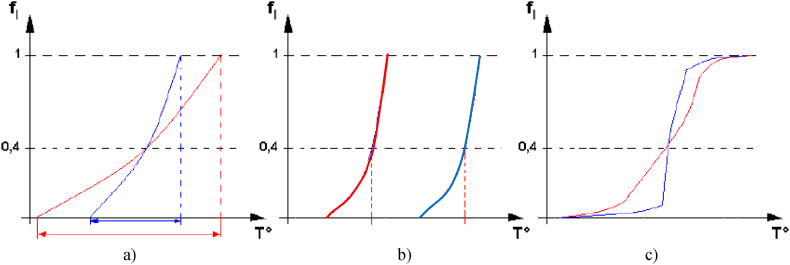
Parameters defining a semi-solid domain suitable for thixoforming: a) amplitude of the semi-solid domain; b) average temperature of the domain; c) Discontinuity or too fast evolution of the liquid fraction. To facilitate thixoforging, the alloy must have a change in liquid fraction vs. temperature that is as close as possible to the characteristics of the red curves. (For interpretation of the references to colour in this figure legend, the reader is referred to the Web version of this article.)

Various criteria are necessary to identify the aluminium alloys as raw materials for a thixoforming process. To choose a particular aluminium alloy as raw material for a thixoforming process, first of all, it is important to know the product characteristics, its expected mechanical properties, the semi-solid interval and the start temperature of melting. Generally, it is necessary to choose the alloy having a semi-solid domain low in temperature and as wide as possible. Thus, the robustness of the heating is increased, and the influence of heat exchange during the forming is reduced. Fig. 1 illustrates some characteristics of the semi-solid domain.

The semi-solid range for a metal alloy, such as steel or aluminium, for thixoforging should ideally correspond to the red curve in Fig. 1, i.e., a large temperature range in Fig. 1(a), a low melting temperature in Fig. 1(b) and have a continuous evolution from the solid to the liquid fraction in Fig. 1(c). Estimating solid/liquid fraction variation with temperature is useful for identifying alloys with suitable compositions for thixoforming. To avoid turbulent flow of semi-solid alloys during the thixoforging processes, the amount of solid phase should not be too low, but neither too high to ensure entire die filling. For thixoforming, the solid phase volume fraction shows values between 30 and 80, but these depend on the forming process [6]. However, in some experimental cases, generally for steel alloys, the solid phase can be higher [7].

Since the rheological behavior of alloys in the semi-solid state is highly sensitive to the solid volume fraction, it is important that the process take place in a temperature interval where the solid volume fraction does not change significantly with temperature variation in order to achieve good thixoformability (i.e., formability in the semi-solid state) [8]**.**

**Fig. 2:**
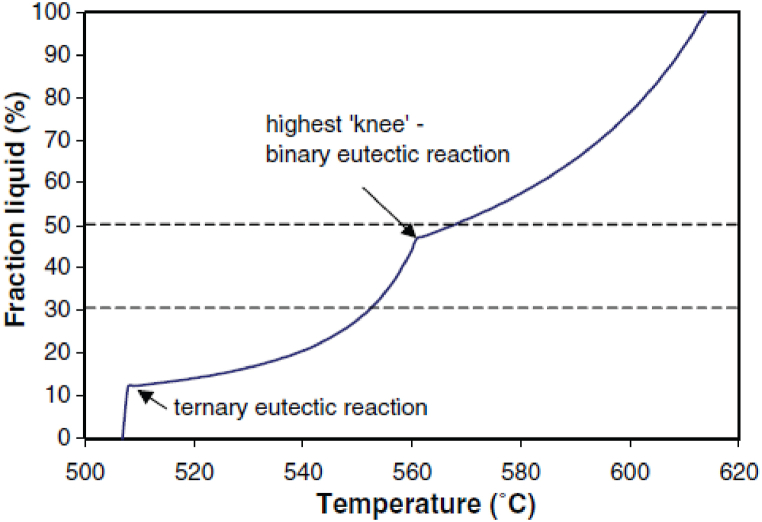
Typical curve evolution of liquid fraction obtains from MTDATA prediction for A356, [11].

The “knee” observed in Fig. 2 on the liquid fraction evolution curve has a “braking” effect on liquid formation and facilitates the process control [9,11]. Achieving favourable results by thixoforging implies a specific amount of solid fraction, which must be adjusted by accurate temperature control during the process. A sufficiently large interval allows the formation (during reheating) of the homogeneous microstructure required for rheological behaviour, favourable to the semi-solid forming [10].

Among the widely used aluminium alloys, foundry alloys have gained researchers in a first step attention due to their good castability and wrought alloys such as the 2xxx, 6xxx and 7xxx series due to their high mechanical properties. For these two reasons, a large number of grades of these two types of aluminium were studied in order to find the appropriate use in conjunction with the thixoforging process. Table A has been compiled using the largest number of significant articles in the literature concerning the thixoforming of aluminium alloys, with their references. In the references presented and according to the articles, aluminium alloys to reach the semi-solid state have been subjected to heating rates that differ from case to case, but are generally between 10 °C and 200 °C per minute [6].

The values of the semi-solid largest interval (ΔT) between solidus and liquidus explored by the authors are presented (Table A) with the mention that it is variable for each aluminium grade, depending on the chemical composition, measurement method, etc. The chemical composition of Al alloys, which was provided by the authors in their studies concerning the thixoforming methodology, shows a slight difference in weight percent of alloying elements. The larger semi-solid intervals make it easier to control the liquid fraction inside the heating system. For the industrial thixoforming of alloys with a narrow semi-solid range (in this case, 6063 aluminium alloy), a globular, refined structure, better equipment, and better temperature control are indispensable [12].

For this reason, it is necessary to choose the right aluminium grade according to the mechanical requirements, the possibility of shaping by thixoforging, the control of the evolution of the semi-solid during the heating cycle (temperature, heating speed, heating mode, etc.) and the specific preparation of the material.

**Fig. 3:**
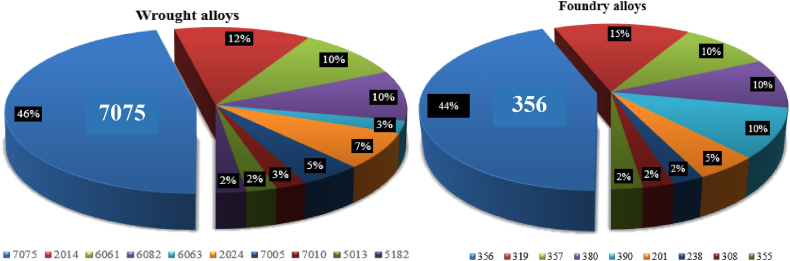
Aluminium alloys processed by thixoforming in the literature.

Fig. 3 summarizes the wrought and foundry aluminium alloys reported in this paper, with the corresponding papers listed in Table A. About half of the papers that investigated wrought alloys studied the 7075-aluminium alloy. Al–Si foundry aluminium alloys such as A365, A319, A357 and A380 raised the interest of investigators and are present in almost three quarters of the papers listed in Table A. The investigation of these materials is closely related to the automotive and aerospace industries, which often require exceptional performance, strength, or heat resistance.

The authors' choice to study one or more grades of aluminium for their work on semi-solid state alloys, their preparation, and shaping, is not always explicit. When this choice is explicit, there are two reasons mentioned:

• the characteristics of the material required for the product
• the thixoformability criteria of the material.

The first point responds to a need in relation to a technical requirement, but in some cases the grade selected is not compatible with a manufacturing process associated with thixoforging. The second point, a suitable selection of the alloy, enables certain criteria to be met to improve thixoforging as shown in Fig. 1. It is the case of wrought aluminium 7075, which has a very large semi-solid range Table A, and moreover, it is widely used in the industry. For this reason, many authors have studied it, as shown in Fig. 3. In the case of 390 foundry aluminium, due to its semi-solid temperature range, it seems to be the most likely to be used for thixoforging. However, it is A356 that has been studied mainly by the authors because it is widely used in the automotive parts production.

It should also be noted that it is not only the semi-solid temperature zone that is of interest, but also other elements of its evolution, which are presented below. Firstly, the methods for obtaining the semi-solid state curves according to temperature are presented, as these can have an influence on the heating and shaping processes.

#### 1.1.1 Evaluation methods of the heating temperature sensitivity

To identify the suitable alloys for thixoforming processing and for the control of this process, the prediction of solid/liquid fraction evolution as a function of temperature is useful. According to the literature, two kinds of evaluation methods can be used: experimental and numerical methods.

• *Experimental methods*

To evaluate the heating temperature sensitivity, the solidus fraction, or the liquidus fraction, the authors used different measurement methods. Generally, to evaluate the semi-solid interval, differential scanning calorimetry (DSC), a thermal analysis technique that measures the variation of the heat flux of melting during the solid–liquid phase transformation, is applied.

**Fig. 4:**
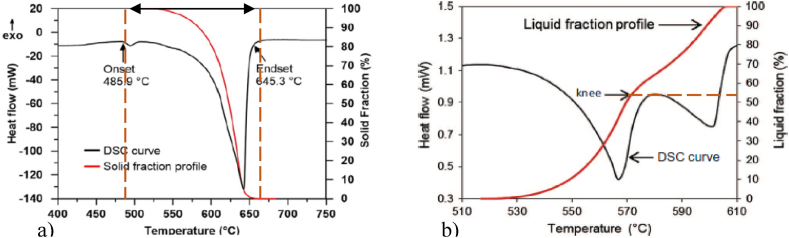
DSC results and: a) solid volume fraction vs. temperature curves obtained by heating 7075 Al alloy samples to 750 °C with a heating rate of 10 °C/min [14]; b) liquid fraction curves for A319 alloy, [13].

**Fig. 5:**
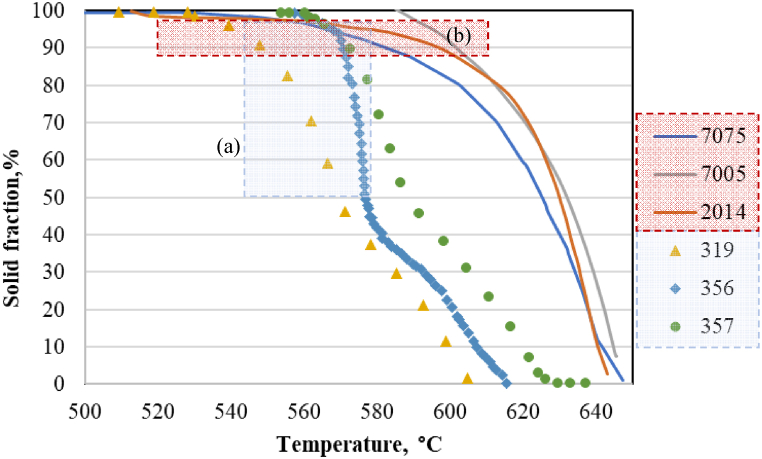
Solid fraction vs. temperature from DSC test at a constant heating rate of 10 °C/min: (3 examples of wrought and foundry aluminium alloys - overlapping curves from the literature).

The solid/liquid fraction variation curves as a function of temperature, shown in Fig. 4, are obtained by integrating the DSC curve. The 7075 wrought alloy has a relatively large melting range of 160 °C compared to the other wrought alloys presented in Table A, and the semi-solid range is around 140 °C (Fig. 4(a)). In the case of A319 cast aluminium alloy, the DSC analysis revealed a “knee” on the liquid fraction vs. temperature curve that occurs at values of 50–60 % liquid, Fig. 4(b) [13]. After this “knee”, the liquid fraction evolves less rapidly with temperature variation. It is for this reason that foundry aluminium alloys are frequently used in rheoforming processes. This knee is observed for foundries aluminium alloys, and Fig. 5 shows three examples.

The shape of the DSC curve is influenced by instrument factors, such as heating rate values, the crucible's atmosphere, the sample location inside the DSC furnace, the thermal resistance between sample and temperature sensor, etc., and also by sample factors, such as the mass, the thermal conductivity, the structure, etc.

For different aluminium grades, Fig. 5 shows some data from the literature collected, in order to evaluate the solid–liquid intervals of the aluminium alloys, performed by standard DSC experiments. Therefore, the comparative diagram shows the variation of solid fraction vs. temperature obtained by DSC testing at a constant heating rate of 10 °C/min for alloys 7075 [14], 7005 [15], 2014 and A356 [9], A357 [16] and A319 [13] commonly studied by thixoforming processes. It can be seen that in the case of the foundry aluminium alloys analysed, the semi-solid domain (50–100 % solid fraction) is narrower, Fig. 5 area (a), and the solid fraction curves are steeper than for the wrought alloys analysed. In the case of the wrought aluminium alloys presented in Fig. 5, area (a), the absolute value of the rate of change of the solid volume fraction decreases as the temperature approaches the solidus (less sensitivity to temperature variation at a high solid fraction), which is not visible in the case of foundry aluminium alloys. The 7075, 7005, or 2014 wrought aluminium alloys show a large solidification interval and are therefore less sensitive to temperature changes and consequently interesting for thixoforming processing. The slope until 50 % solid fraction is very steep for these alloys, but it changes then and is relatively flat for 2014 and 7075 at high solid fraction values, which may provide a wider processing range for thixoforming.

It should be noted that the ramp rate and test sample mass have an impact on the DSC test results. This is so because the temperature that is being measured is not the sample temperature rather, it is the reference temperature [17].

The failure of semi-solid processes is mainly caused by the difficulty to control the process parameters, but also by the lack of quantitative criteria [8]. Tzimas and Zavaliangos [8], for instance, introduce a simple criterion for binary eutectic alloys based on the sensitivity of the volume fraction of the solid depending on variations in temperature. The Single Pan Scanning Calorimeter (SPSC) allows better accuracy in measuring enthalpy data, thus avoiding the smear effect in DSC experiments [17]. The temperature sensitivity of an alloy designed for semi-solid processing (SSP) is suggested to be as low as possible [9]. For 7075 alloy, according to the results obtained by DSC analysis regarding the evolution of the solid fraction as a function of temperature, it was observed that a solid fraction between 0.9 ÷ 0.7, necessary to obtain a thixotropic behaviour can be obtained in the semi-solid working temperature range of 580–610 °C [18]. This solid fraction range provides a good achieve process.

Differential thermal analysis (DTA) has been applied by researchers to determine the appropriate thixoforming temperature range for commercial aluminium alloys. Chen and Jeng [19] note the advantages of the DTA method compared to conventional thermal analysis due to the fact that it is more sensitive and reliable.

The amount of solid/liquid phases is also estimated by quantitative metallography on the prepared metallographic surfaces of quenched samples for different temperatures of semi-solid slug and so for different liquid fraction. Neag et al. [20] studied the microstructure characteristics by Image software. Gu et al. [21] used Image software to analyse ex situ the reconstructed volumes from micro X-ray micro tomographic slices. They obtained a good concordance between 2-D SEM image analyses and 3-D X-ray microtomography for M2 steel.

In situ techniques, which are not destructive, are increasingly used and preferred to study the evolution of the liquid fraction and the microstructure at the semi-solid state but with dimensional limits as a micro slug. Terzi et al. [22] observed, using in situ X-ray microtomography the events that occur during partial remelting of a cold-rolled Al–8 wt.% Cu alloy.

High-temperature Confocal Laser Scanning Microscopy (CLSM) has also been used by Gu et al. [21] to perform in situ studies of microstructure evolution during both reheating to the semi-solid state and cooling to room temperature, respectively.

Selecting the evaluation methods to investigate the temperature sensitivity and the efficiency of a desagglomeration technique during reheating would be very interesting. Even though high-energy X-ray microtomography [21,23] or CLSM are powerful tools, they are not as easy to use, and optical and scanning electron microscopes of the specimen after completion of solidification (quantitative metallography) are still more accessible in laboratories.

• *Numerical methods*

**Fig. 6:**
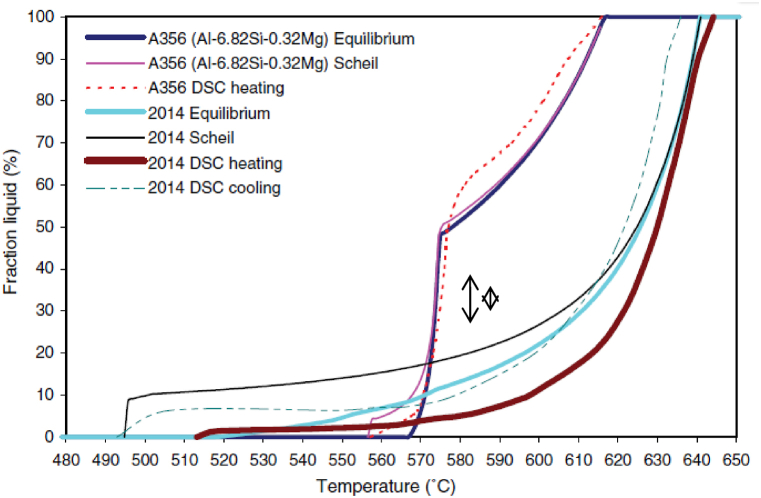
Fraction liquid vs. temperature from MTDATA and DSC (rate 10 K/min) for as-cast alloy A356 (Al–6.82Si–0.32Mg–0.022Cu–0.005Zn–0.112Fe–0.1Ti–0.013Pb–0.042Sn–0.006Ni–0.005Cr, wt.%) and as-extruded alloy 2014 (Al–3.91Cu–0.47Mg–0.83Si–0.29Fe–0.55Mn, wt.%). The MTDATA calculations for A356 are for a simplified alloy: Al–6.82Si–0.32 Mg wt.%. For 2014, the composition for the MTDATA calculation is the same as for the DSC sample [9].

**Fig. 7:**
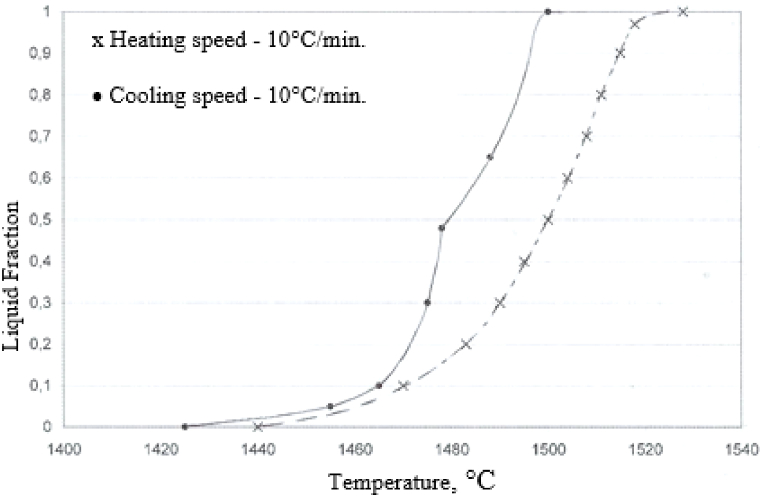
Evolution of the liquid fraction vs. temperature from DSC (rate 10°/min) for C38 steel.

A few numerical methods are applied to identify a reference for the experimental curves. Tzimas and Zavaliangos [8] estimate the processability (thixoformability) in the semi-solid state of some simple binary alloys (such as Al–Mg, Al–Cu, Al–Si, for example) using DSC analysis and analytical formulae (lever rule and Scheil equation). They introduce a first order criterion applied in order to evaluate binary alloys’ processability in the semi-solid state. This criterion is based on the sensitivity of the volume fraction of solids to temperature variations. Camacho et al. [24] proposed as a guide for quantitative study of alloying elements effect on the evolution of the liquid fraction during Al–Zn–Mg, Al–Zn–Mg–Cu systems or commercial multicomponent alloys solidification, an MTDATA thermodynamic prediction software package. In addition, Liu et al. [9] estimated by MTDATA the impact of copper content on the thixoformability of A356 aluminium alloy and the impact of silicon content on the thixoformability of 2014 aluminium alloy. They assumed Scheil type conditions during thixoforming, justifying this by the fact that when induction reheating is applied, the semi-solid state is reached rapidly, and therefore, the time to establish equilibrium is insufficient. MTDATA modelling investigation results of liquid fraction sensitivity are useful compared to DSC because they do not use samples, therefore avoiding long experimentation times. However, MTDATA does not consider routes for obtaining the semi-solid alloy: by cooling associated with rheoforming (usually a large liquid fraction) or by partial melting associated with thixoforging. Fig. 6 shows DSC heating results for foundry alloy A356 and wrought alloy 2014 comparative with Scheil and equilibrium calculations. For the foundry alloy A356, the difference between the experimental and the modelling is small, with a maximum deviation of 10 % of the liquid fraction for a liquid fraction range between 60 and 95 %. For wrought alloys, however, the differences are greater, especially for the comparison with the DSC result in the heating phase. It is more representative with a DSC result in the cooling phase. At the same temperature, the 2014 aluminium alloy shows a higher quantity of liquid phase during the cooling phase, which was also observed in the case of C38 steel by ASCOMETAL CREAS (see Fig. 7).

Zoqui et al. [25] noted that experimental thermodynamic characterization using DSC, differential thermal analysis (DTA) and Thermo-Calc® simulation (based on the Scheil model for non-equilibrium solidification) are efficient in predicting the behaviour of semi-solid materials since they identify the transitions to be avoided, i.e. eutectic reaction and complete melting.

The size of the samples for the DSC, the DTA, or the crucible is of the order of mm^3^. In the case of larger parts and depending on the heating mode, there may be temperature gradients in the volumes, and consequently, the semi-solid is heterogeneous. Therefore, for the prediction of the solid/liquid fraction vs. temperature required to control the thixoforming process, it is preferable to consider both the effect of semi-solid transformation kinetics, thermodynamic factors, and the type of raw material and to use experimental or numerical methods for evaluation.

#### 1.1.2 Chemical composition influence on the solidification interval

There are studies in which the improvement of thixoformability was achieved by varying the chemical composition, which has effects on the semi-solid range. For example, by adding up to 10 wt% copper, the working window of A356 aluminium alloy enlarge to between 30 % and 50 % liquid fraction [9]. Also, for 2014 aluminium alloy, the addition of silicon increase the amount of eutectic and decrease the solidification interval, which improves the thixoformability and decreases the tendency for porosity formation [9].

The very steep slope of the liquid fraction vs. temperature (under 50 % liquid) observed for foundry aluminium alloys, which is steeper than that for forged aluminium alloys (see Fig. 5, Fig. 6), implies that the latter are more suitable for thixoforming. The characteristic of foundry aluminium alloy A356 is the formation of a distinctly “knee” at about 40 % solid [9]. Observations reported in the literature point out that the solidification range must be less than 130 °C for the 2014 alloy, due to its limit of thixoformability and susceptibility to hot tearing, and of 71 °C for A356 aluminium alloy [9].

Tzimas and Zavaliangos [8] estimated by DSC that since alloys such as A3003 and A6061 have small variations in the semi-solid interval and respectively, a large variation of the solid fraction, these alloys cannot be thixoformed. They also observed that an Al–Mg alloy with a composition <7 wt% Mg is too sensitive to temperature variations to be produced via thixoforging processing routes. Unpredictable changes in the rheological behaviour of the alloy that will impact the final properties of the parts can thus be avoided.

**Fig. 8:**
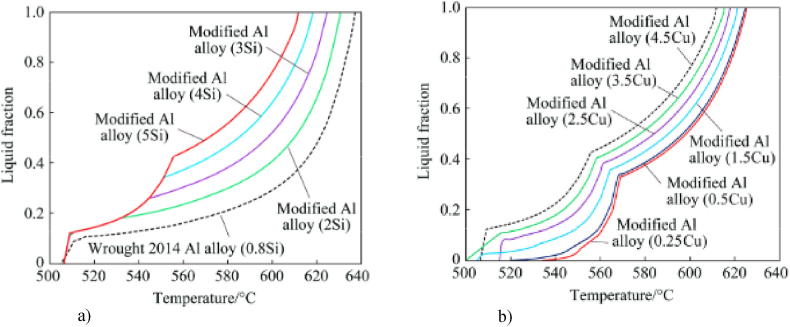
Liquid fraction vs. temperature for wrought 2014 Al alloy and 2014 alloy modified (Al–5Si− 4.5Cu−0.5Mg−0.8Mn−0.7Fe−0.25Zn): a) with different amounts of Si; b) with different amounts of Cu, calculated using JMatPro simulation software [26].

Arif et al. [26] studied the wrought 2014 aluminium alloy thixoformability by a numerical method and evaluated the effects of the alloying components on the evolution of the liquid fraction during solidification. As illustrated in Fig. 8(a), increasing Si content in wrought 2014 Al alloy has an decreasing effect of the solidification interval and the amount of eutectic phases rose, as shown by Liu et al. [9]. As shown in Fig. 8(b), the reduction in the Cu content allows to a decrease in the solidification interval temperature of 2014 aluminium and the amount of eutectic phases (the highest “knee” point drops), as Liu et al. [9] predicted for A356 aluminium alloy. Arif et al. [26], also noted that increasing in Mg content has no important effect on the amount of eutectic phases nor on the solidification interval, but the formation of compact π-Al8FeMg3Si6 phase and decrease in the amount of sharp and plate-like structure of β-Al5FeSi phase improve the strength of the modified alloy.

#### 1.1.3 Thermal kinetic effects

Many researchers focused their efforts on studying the solidification material behaviour. The current evaluation methods can only predict solidification phenomena, which is difficult when thermal kinetic effects are significant. The kinetics effects during reheating should be considered to reach the desired temperature and to have a good control of the process. In this context, Zoqui et al. [25] noted that even if thermodynamic characterization using DTA, DSC and Thermo-Calcs® simulation are efficient in predicting the behaviour of semi-solid materials, using only these criteria can reduce the number of alloys considered adequate for semi-solid state processing. For an aluminum alloy system, it is recommended that the sensitivity of the liquid fraction df_L_/dT at the 0,4 liquid fraction be as low as possible (less than (<0.03 °C^-1^) [9]. Zoqui et al. [25] adopted for the primary phase the sensitivity of the liquid fraction df_L_/dT less than 0.03 °C^-1^ and noted that even lower sensitivity could be used. They suggested that this criterion will allow increasing the number of thixoformable alloys by focusing on the control of the semi-solid processes.

**Fig. 9:**
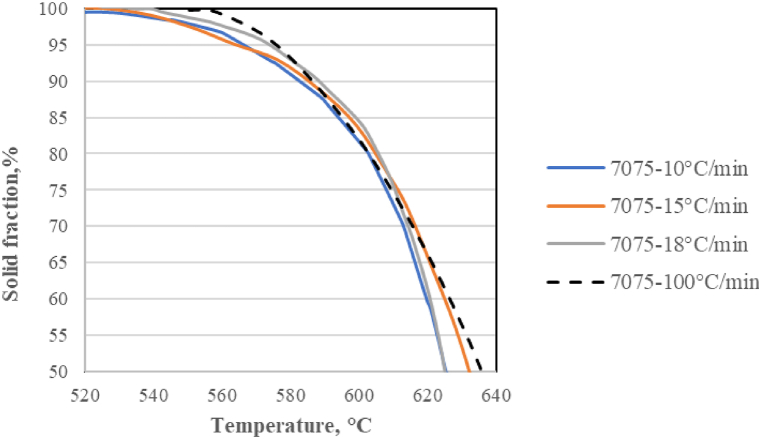
Solid fraction vs. temperature from DSC test at different heating rate of 7075 aluminium alloy.

Nafisi et al. [27] examined the evolution of the solid fraction for Al–Si alloys using quantitative metallography, computer-aided cooling curve analysis, and computational thermodynamic methods. They concluded that the solid fraction is overestimated as determined by the quantitative metallography.The main explanation for the observed differences is the shifting of the eutectic and liquidus lines, which are linked to higher cooling rates. It is important to note that generally the heating rate used in DSC or DTA and presented in the literature is slow (between 10 and 18 °C/min respectively), and the equilibrium conditions are considered, while under experimental conditions higher heating rates are achieved. So, an important challenge for the evaluation methods is the heating rate, which under industrial thixoforming conditions is around 50–100 °C/min (inductive, but also conventional radiation/convection-type heating). In this way, the solid fraction evolution vs. the temperature for the 7075 aluminium alloy, obtained by DSC constant heating rate of 100 °C/min was compared in Fig. 9 with the results collected from the literature, where the tests have been conducted at heating rate values of 10 °C/min [28], 15 °C/min [18], and 18 °C/min [29] as reported.

The solid fraction evolution is obviously influenced by the heating rate. At the faster heating rate, the solid contents of the semi-solid slurries are slightly higher, which means sensitivity and productivity increase. The result is that heating curves evolve toward higher temperatures when the heating rate increases, but this is not obvious throughout the temperature range.

**Fig. 10:**
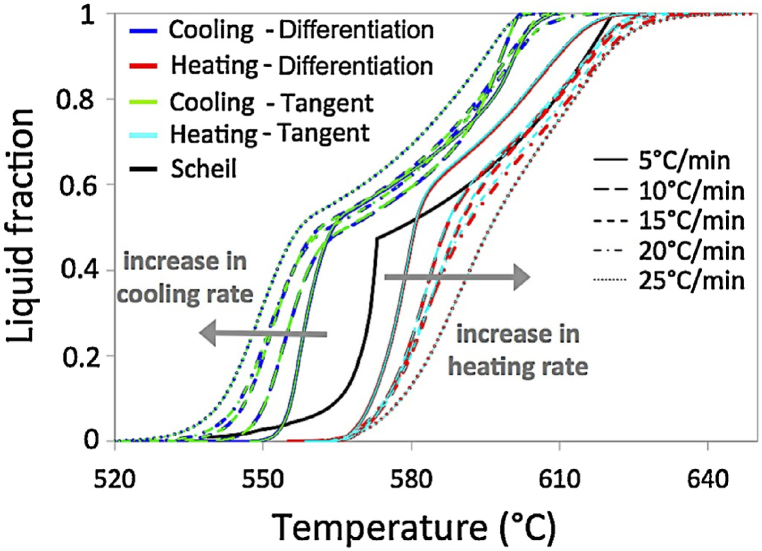
Temperature-dependent liquid fraction curves for A356 alloy generated by integrating the partial areas under the cooling and heating DSC curves. As a reference point, the curve predicted by numerical simulation (Scheil condition), [30].

Brollo et al. [30] explained the relative positions of the A356 alloy curves obtained by the DSC method (see Fig. 10). According to their theory, phase transformations are delayed and occur over a wider temperature range when cooling or heating rates are higher. This results in a larger driving force for melting, which causes the phase transformations to begin earlier. Accordingly, the temperature range in which the diffusion processes take place is greater than that which equilibrium conditions would predict. A similar trend was later noted by Brollo et al. [31] with reference to the impact of heating rate on temperature range for aluminum alloys A355 and B319.

**Fig. 11:**
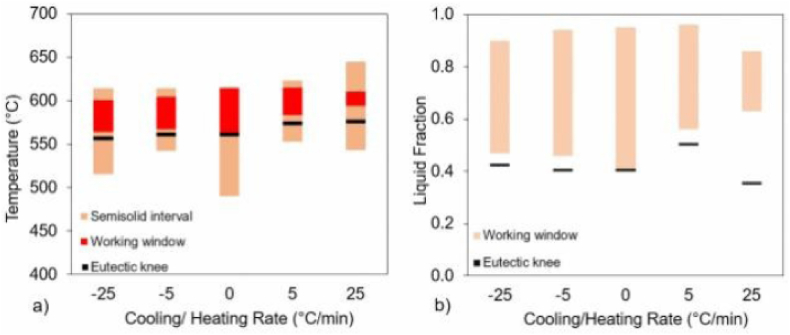
Evolution of thixoforming process parameters for Al5Si5Zn0.5 Mg alloy under various kinetic scenarios: temperature (a) and liquid fraction (b). The Scheil condition is shown with a heating rate of 0, and cooling rates are represented by negative heating rates; [32].

Rodrigues Dantas et al. [32] used numerical simulation accomplished under the Scheil condition and applied the differentiation method to DSC data to estimate the thermal stability of the Al–Si–Zn–Mg alloy and to identify the thixoforming working range, respectively. Fig. 11 exhibit for Al5Si5Zn0.5 Mg alloy the thixoforming process parameters obtained under different kinetic conditions. Rodrigues Dantas et al. [32] observed that the cooling rate changes little the liquidus temperature, while the heating rate has a more significant effect on the liquidus temperature in the studied range, due to the reaction inertia. They offer the same explanation for the solidus temperature decreases with increasing the cooling rate. Also, during the high heating rate regime, the melting of the intermetallic phases was associated with a higher amount of energy as a function of time, which facilitated the decrease in the temperature of the solid. Moreover, at higher heating/cooling rates, larger semi-solid intervals can be observed with only a slight influence on the corresponding knee temperature.

**Fig. 12:**
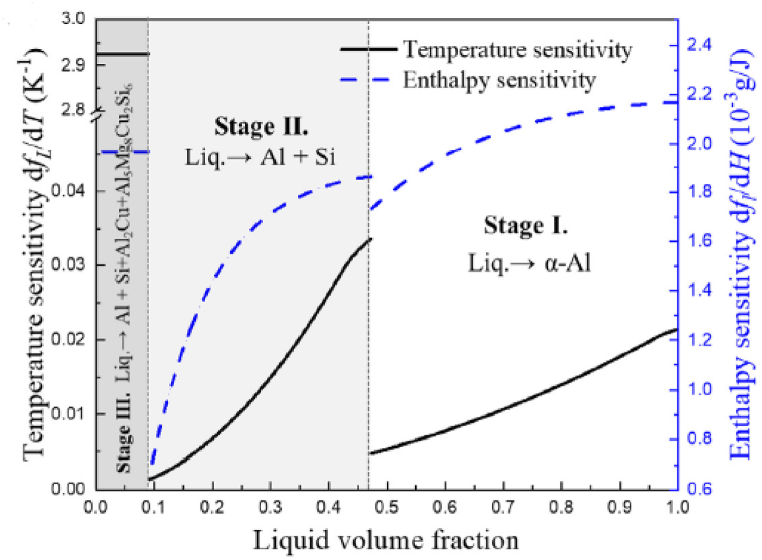
Enthalpy and temperature sensitivity of liquid volume fraction based on single-pan scanning calorimeter measurements of 319 s, [34].

Many authors view the slope of the liquid fraction curve (df_L_/dT), also known as the process time sensitivity (df_L_/dT), as a crucial factor in determining how easily processed semi-solid materials are. Hu et al. [33] proposed an enthalpy sensitivity criterion for assessing the processability of semi-solid alloys. They noticed that the semi-solid's processability at 0.4 liquid fraction is significantly better than 0.7 liquid fraction based on enthalpy sensitivity. (Fig. 12).

When selecting materials for thixoforming, characterisation of the melting range and prediction of the influence of thermal parameters in the semi-solid state are essential. Thixoformability can be significantly improved by using an aluminum alloy with a wide semi-solid temperature range and a continuous evolution from the solid to the liquid fraction.

To choose an aluminium alloy as raw material for a thixoforming process, first of all, it is important to know the product characteristics, his mechanical properties expected and secondly the semi-solid interval and the start temperature of melting.

During the semi-solid forming, the solid fraction is a critical parameter. It is a known fact that the content in alloying elements can play a significant role on the solidification interval temperature. Different measurements or calculations methods can be used to estimate the temperature sensitivity for both a cast alloy composition and a wrought alloy composition. To evaluate the semi-solid interval, the experimental method of differential scanning calorimetry is mainly used. For a given alloy, both the solid/liquid fraction curves at low and high heating rate must be respected in order to approximately represent industrial conditions. The most suitable alloys for thixoforming can be identified based on the prediction of the evolution of the solid/liquid fraction.

### 1.2 Semi-solid material preparation for thixoforging

It is well known that a material's microstructural characteristics generally affect how well it performs, and that this also holds true for how well the semi-solid forming technology performs. In the semi-solid processing, it is preferable to use a non-dendritic microstructure alloy, i.e. a suspension of solid, nearly globular primary particles surrounded by liquid matrix necessary to provide a thixotropic flow [5]. This was also pointed out by Tzimas and Zavaliangos [8] in their studies concerning the aluminium alloys and by Bigot et al. [35] on Sn-15 % Pb alloys.

#### 1.2.1 Methods for preparing the semi-solid billets

**Fig. 13:**
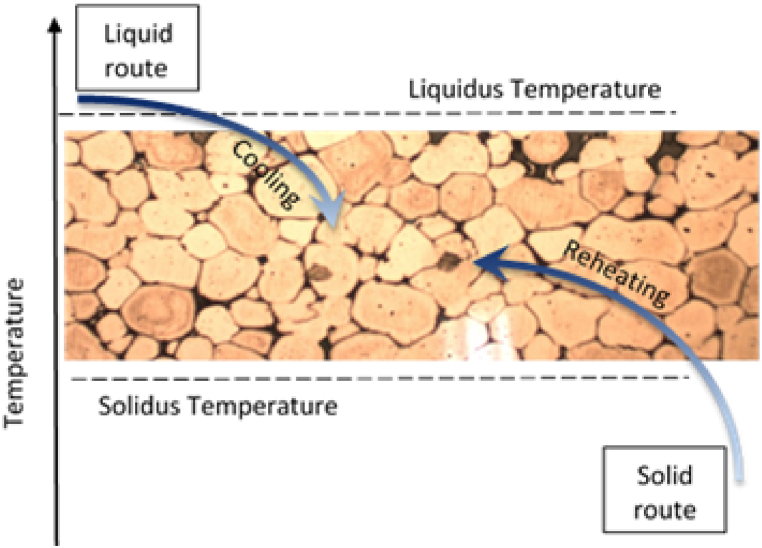
Schematic illustration of different routes for Thixoforming.

Several methods to produce the semi-solid billet, generally divided into liquid and solid route (which are displayed in Fig. 13) that allow to obtain a non-dendritic microstructure for thixoforging processes have been proposed over the years on the scientific studies [3,4,36].

The overheated molten alloy is brought to a semi-solid temperature via the liquid route via mechanical stirring, electromagnetic stirring, ultrasonic stirring methods, chemical grain refining, spray casting [3], shearing-cooling roll [37], swirled enthalpy equilibration device process [38], gas-induced semi-solid process [39], electromagnetic stirring combined with mechanical vibration as mentioned Dao et al. [40], cooling slope casting [41], low superheat casting [42] and several more and can then be thixoformed.

The solid route can also be used to obtain the required aluminium non-dendritic microstructure. The solid route, applied to produce a non-dendritical fine microstructure during the subsequent reheating process, is defined by the material's thermo-mechanical history. An important advantage of the solid route is due to the fact that many aluminium alloys are delivered in an extruded or rolled state, with a non-dendritic microstructure that gives better flow than those produced by liquid state routes to thixoformable microstructures (e.g., magneto hydrodynamic stirring) [[2], [43]]. The most popular solid state routes for semi-solid material preparation are: recrystallization and partial melting (**RAP**) [36], strain induced melt activation (**SIMA**) [44] and semi-solid thermal transformation (**SSTT**) [45] or wrought aluminum directly semi-solid isothermal treatment (WADSSIT) [46].

The RAP method and SIMA method are most frequently applied to obtaining aluminium alloy characteristics for semi-solid forming. Before semi-solid forming, a reheating step is necessary in both cases to spheroidize the solid grains in order to increase the alloy fluidity [47]. The methods can be applied both to the aluminium foundry alloys and wrought aluminium products, using simpler equipment as compared to the means applied during cooling (e.g., mechanical stirring) by the liquid route.

*The RAP method* adopted by Bolouri et al. [48] is based on the first stage on working the material at temperatures below the recrystallization temperature (for example, by warm extrusion) to induce sufficient strain energy in the microstructure, which has an effect on the evolution of globular grains in the semi-solid state. Prior to being thixoformed, the billet that was cut from the extruded bar is reheated to a semi-solid state in the second stage. Atkinson et al. [49] explained that when the material is in the semi-solid state, recrystallization happens and the liquid phase penetrates the recrystallized boundaries, producing equiaxed grains well rounded in a liquid matrix, as Neag et al. [18] also observed. After the desired semi-solid temperature is reached in the whole billet volume, the thixoforming process will be conducted under the conditions of a laminar flow of the semi-solid material. Most of the literature results concerning the RAP route applied to preparing the semi-solid microstructure are about the 7075 aluminium alloy, but there are studies also on the 7005, 6061, 2014 and 2024 alloys, as well as on foundry alloys, of which we mention A356 and A380.

*The SIMA method* [44] is based in the first stage on working the material at temperatures higher than the recrystallization temperature. This is followed by quenching or cold deformation (to induce enough strain) before reheating the alloy to a temperature within the solid-liquid temperature range. The critical parameters for controlling the desired semi-solid microstructure in the SIMA process include: the initial degree of cold working and the heating parameters. Researchers have turned to the SIMA route to investigate the behaviour of various aluminium alloys, including 7075, 7005, 5013, 2014, A356, and A380 alloys.

Bergsma et al. [45] mention *the SSTT method* applied for a dendritical structure with an initial grain size that is too large (unsuitable for semi-solid production) that supposes a rapid induction heating and isothermal maintain for a short period of time at the semi-solid temperature, to produce a spheroidal structure. The heat treatment applied to produce a spheroidal microstructure suggests a suitable rheology for semi-solids forming over a short period of time and is considered an effective alternative to industrial production. The wrought aluminium directly semi-solid isothermal treatment (WADSSIT) process provided optimum conditions for semi-solid aluminum alloy billet preparation [46] as well as the semi-solid isothermal treatment of hot extruded alloys (SSITHEAA) [50].

#### 1.2.2 The influence of extrusion ratio

The suppliers of extruded aluminium alloys deliver bars with different extrusion ratios and different diameters. Over time, feedstock materials with various extrusion ratios have been studied.

**Fig. 14:**
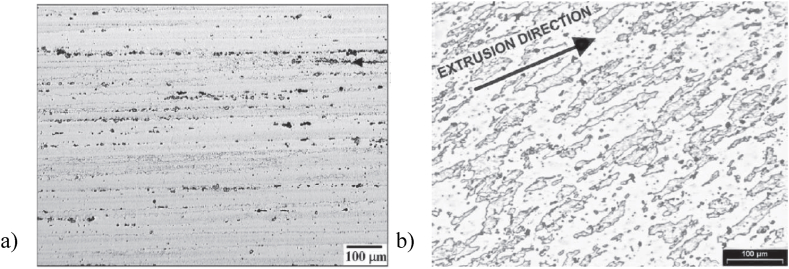
As extruded microstructure of the wrought 7075 alloy with an extrusion ratio of 16:1 from different suppliers; a) 64 mm bar [29]; b) 40 mm bar [18].

Chayong et al. [29] studied a commercially extruded bar (i.e., adopting the RAP route) with an extrude ratio of 16:1 and T6511 treated, which shows intermetallic particles aligned in the extrusion direction (see Fig. 14(a)) and demonstrated that 7075 aluminium alloy can be thixoformed and successfully fill the die. In addition, Neag et al. [18] explored an extruded 7075 aluminium alloy (i.e., they adopted the RAP route for a bar with an extrusion ratio of 16:1 and a T6 heat treatment) see Fig. 14(b) and noted that after reheating this alloy to a semi-solid state, a homogeneous microstructure of globular solid grains surrounded by a thin liquid film (see section 3.3) is formed, which is ideal for thixoforming.

Jiang et al. [51] strengthen the studies concerning the evolution of the 7075 aluminium alloy microstructure and promote the application of the thixoforming process using a bar with a lower extrusion ratio of 9:1, and furthermore note that the microstructure obtained by the RAP method offers a better spheroidization efficiency compared to the SIMA microstructure. They show RAP samples extruded at 300 °C and directly used in the semi-solid isothermal treatment. The comparison was made with SIMA samples extruded at 400 °C, upset at ambient temperature (pre-strain of 12 %) and then used in the semi-solid isothermal treatment. Mohammadi et al. [52] obtained a thixoformable microstructure of 7075 alloy using a feedstock material extruded at 420 °C with a larger extrusion ratio of about 20:1.

**Fig. 15:**
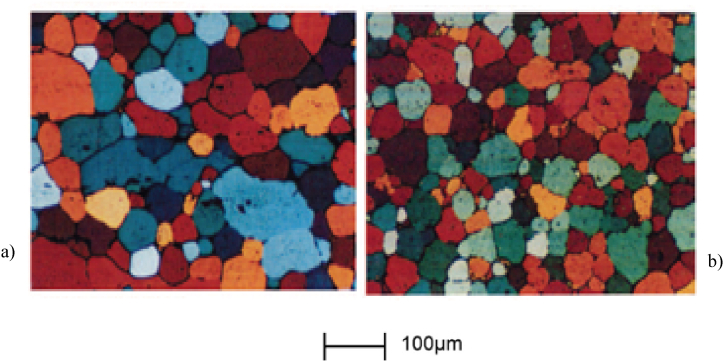
Micrographs of 7075 aluminium alloys (sample dimensions 2.5 × 10 × 15 mm) with an extrusion ratio of: a) 8.5; and b) 16; heated for 10 min in a salt bath at 582 °C and quenched in brine [49].

Atkinson et al. [49] evaluating the influence of extrusion ratio (Fig. 15 (a) and (b)) on the partially remelted microstructure of 7075 aluminium alloy after 10 min in a salt bath and quenching in brine, came to the conclusion that, the higher the extrusion ratio, the greater the overall grain boundary and sub-grain boundary area and greater texture development in the starting material. This allows for a greater potential to develop recrystallized nuclei and thus produces a finer size of the recrystallized grain (Fig. 15 b). Also, they noted that, the misorientation of the subgrain boundaries may increase with an increased extrusion ratio, leading to an increased amount of stored energy in the material.

Additionally, Sang-Yong et al. [53] observed that for SIMA-prepared 7075 alloy, at least 50 % cold working (by swaging) is necessary in order to obtain uniform and fine globular microstructures of (about 30 μm) after billet induction reheating to a semi-solid state, and the holding time should not exceed 3 min in order to prevent grain growth. Hassas-Irani et al. [54] noted that for A356 aluminium alloy fabricated by the SIMA route, a 45 % pre-strain obtained by compression of the heated specimens in an electrical resistance furnace up to 615 °C provides the most desirable microstructure for the subsequent thixoforming process.

**Fig. 16:**
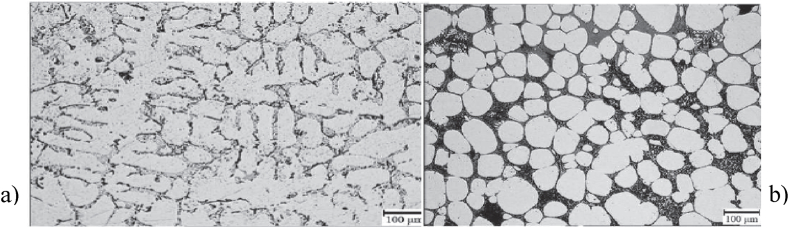
Microstructures of A356.2 alloy: (a) as-cast and (b) 30 % compression ratios after isothermal holding at 580 °C for 10 min [55].

Yan et al. [55] mentioned that the desirable microstructure for the subsequent thixoforming process can be obtained from a 60 % cross wedge rolling pre-formed sample (to induce large strain and good deformation uniformity of the specimens) if an isothermal treatment is applied and no more than 30 % compression ratio in the same direction as the rolling direction (Fig. 16).

Bolouri et al. [48] examined the microstructure of the aluminium 7075 alloy in the semi-solid state using SIMA processing and discovered that the nucleation rate increases more quickly as the amount of deformations (compression ratios) increases. A decreasing trend in the mean α(Al) grain diameter and an enhancement in spheroidization were noted in conjunction with an increase in the alloys' compressive ratio.

#### 1.2.3 The influence of reheating parameters on the partially remelted microstructure

One of the essential steps of thixoforging process consists in the billet reheating, which can be influenced by a variety of factors. From an industrial standpoint, it is important to deform at high solid fraction values (i. Low energy consumption for reheating; ii. A certain consistency that allows optimal conditions handling), but this may limit the benefits of thixoforming. If the reheated billet contains a lower solid fraction, there is a risk of distortion of the semi-solid shape before the deformation stage, which will influence die filling or even a much stronger macro-segregation of the liquid. Component quality in terms of shape and surface finish may suffer from macro-segregation. On the deformation stage, the liquid segregation phenomenon can affect the microstructure and therefore the mechanical characteristics of the thixoforged component.

Although there are no test standards appropriate for evaluating aluminium alloys in the semi-solid state, there are a number of experimental findings that have been published in the literature. The final part's microstructure, mechanical properties, and semi-solid material flow are all influenced by the reheating parameters.

**Fig. 17:**
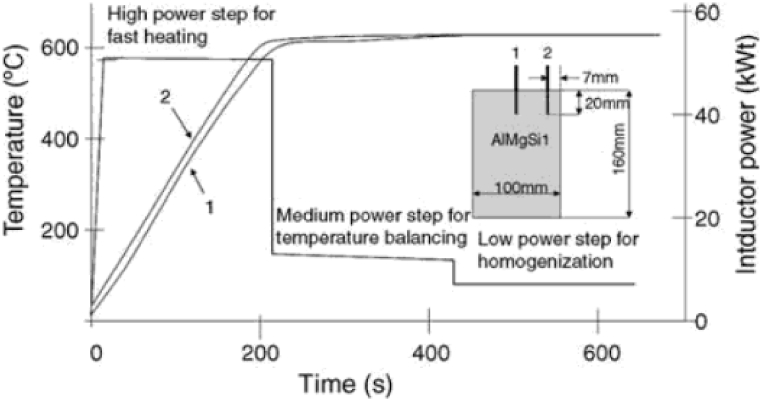
Typical heating cycle for AlMgSi1, [6].

**Fig. 18:**
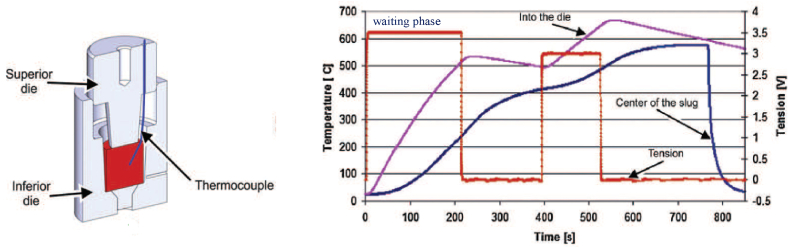
Induction heating cycle of 7075 aluminium alloy: “K” type thermocouples measure the temperature [18].

The reheating temperature, the amount of time held at a constant temperature to achieve an isothermal temperature profile, the reheating rate, and the number of reheating steps are all considered reheating parameters, which vary based on the heating system. The reheating parameters depending on the heating system, are the reheating temperature and the holding time at a constant temperature, in order to reach an isothermal temperature profile, as well as the reheating rate and the reheating steps, as can be seen in Fig. 17 or Fig. 18. To obtain a fine, homogeneous, and globular structure, the processing parameters, including the reheating parameters, must be selected properly. Researchers reported generally using induction or convective heating (electric resistance furnace)regimes to reach the semi-solid temperature necessary to complete the globularisation, and Table 1 summaries that. Both heating systems had benefits and drawbacks.

**Table 1:** Summary of heating systems used to reach the semi-solid state.

Heating System	Authors	Aluminium alloys
Induction heating	[11,13,18,29,51,56,[60], [61], [62], [63], [64], [65], [66], [67], [68], [69]]	A356, A356, A390
		7075, 7005, 2024, 2014
Convective heating	[18,28,33,48,50,52,54,55,57,73,[81], [82], [83], [84], [85]]	A356, A380, A319,
		7075, 6061, 2024
Salt bath	[49,58,59]	201, 2014, 7075

#### 1.2.4 Induction heating regimes

Reheating the billet or the system containing the billet by induction allows faster heating and provides the possibility of flexible process control [57]. Depending on the chosen heating frequency, the billet is heated in a localized manner in a thickness of the volume, generally radially. If the billet is large, the heterogeneities increase. In the case of induction heating, to obtain a homogeneous temperature for the billet, it is necessary to alternate heating and waiting phases. During the waiting phases, the core of the billet continues to be heated through conduction from the exterior surface. The exterior surface of the billet is cooled by heat conduction towards the core as well as convection and radiation with the external environment. Considering these conditions, the liquid fraction is not homogeneous due to the temperature evolution, which is influenced by the billet geometry and the heating cycle parameters. To reduce the skin effect, characterized by the so-called penetration depth, low frequency heating can be applied if only the homogeneity of the temperature distribution would have to be considered. But for rapid reheating, typical heating cycles are required, which consist of several stages until the surface of the billet reaches the target temperature [57].

Induction reheating systems used to achieve a semi-solid state (i.e., the target temperature) are applicable to both the wrought and foundry aluminium alloy families. Some examples from the literature where researchers have reported using induction reheating systems to reach the semi-solid state are listed in Table 1. The billet can be reheated to the semi-solid state directly by induction or by placing the billet into the die to avoid the convective and radiation heat losses during the transfer from the furnace to the press [6].

Chayong et al. [29] found that after three-step rapid induction heating of 7075 aluminium alloy in the range of 620 °C, the microstructure displays fully recrystallized grains, and if the hold time is brief, microstructural coarsening is inhibited. Chayong et al. [58] studying the 7075 aluminium alloy, revealed more detailed effects of one-step, two-step, and three-step induction heating regimes in this case. Furthermore, Atkinson and Liu [59] reported that 7075 is very resistant to recrystallization in the solid state, which they attributed to the presence of dispersoids pinning grain boundaries.

Some published papers propose a three-step [6] induction heating regime to heat AlMgSi1 billets to a semi-solid state (Fig. 17). In the first step of heating, a high power is applied (180°/min until the surface reaches the semi-solid temperature), after which the power is reduced (to compensate for the heat losses due to convection and radiation), and on the last step, a low power is applied to allow billet temperature homogenization (by heat conduction).

Neag et al. [20] heat the billet inside the die used for thixoforming, to achieve isothermal conditions. In their study, it is suggested 4 successive induction heating power stages (see Fig. 18) with an average reheating rate of about 45 °C/min, to obtain a homogeneous semi-solid temperature in the 7075 aluminium alloy.

In the case of 2014 aluminium alloy, three-step induction heating at 627 °C (highinitial heating rate of about 237 °C/min) allows to obtain a microstructure primarily composed of spheroidal grains with occasional unrecrystallised elongated grains retained (associated to inhomogeneous dispersoid pinning on the grain boundaries or inhomogeneous strain) [11]. Jung et al. [60] recommended a relationship that allows the identification of the optimal design of an inductive coil used for uniformly heating the billet to attain the semi-solid state. For A356 billet, they proposed a three-step reheating process, to obtain fine globular microstructure. They noticed that a globular microstructure cannot be obtained if the last reheating step is too short, and that the risk of coarse grains may increase if the holding time is too long. Kang and Seo [62] also reported a three-step induction reheating process for the alloys A357 and 86S, which allows to obtain a uniform temperature distribution in the billets with a homogeneous and fine globular microstructure.

Jiang et al. [51] observed that increasing holding time in the semi-solid state led to coarsening of the RAP and SIMA, respectively, and increasing isothermal temperatures to 600 °C led to an increase in grain size (by the effect of melting and coarsening of solid grains).

Zhang et al. [70] observed that, using an intermediate frequency electromagnetic oscillation process to prepare AlSi9Mg aluminium alloy semi-solid slurry, after a reasonable number of oscillation periods, a fine, globular, and homogeneous microstructure could be obtained.

#### 1.2.5 Convective heating regimes

Convective heating systems like induction heating systems are used to achieve a semi-solid state (i.e., the target temperature) for both wrought and foundry aluminium alloy families.

Some examples from the literature where researchers have reported using convective heating systems to reach the semi-solid state are listed in Table 1. Reheating the billet or the system containing the billet using a convective heating regime achieves an accurate and uniform temperature, but the heating time is much longer.

Mohammadi et al. [52] used a resistance furnace to reheat the 7075 aluminium alloy (previously extruded at 420 °C with a higher extrusion ratio of 20:1) at 580 °C with an average heating rate of 1 °C/s. They concluded that the formation of spheroidal grains was accelerated by a high semi-solid isothermal temperature (80 % solid fraction ranging). Bolouri et al. [48] examined the effects of predeformation rate on the microstructure evolution of cold compressed 7075 Al alloy samples heated at a semi-solid state in a convective heating furnace. They noted that on high solid fractions (>70 %), extending the holding time (in this case to 60 min), facilitates the increase in the degree of spheroidization, but also the grain growth and the thickness of the grain boundary. Neag et al. [18] heated the billet inside the steel die to avoid heat losses until forming and observed that the liquid fraction reaches its steady state after 30 min in a convective heating (resistance heating furnace) (Fig. 18). Also, the grain boundaries are better lubricated by extending the holding time, which stimulates recrystallization and spheroidization in the 7075 extruded aluminium alloy.

## 2 Discussion

For industrial applications, the heating strategy is very important, and the heating control is essentially everything. The reheating regime affects the microstructure and flow behaviour of the material during semi-solid forming and may influence the mechanical properties of the thixoformed part.

The convective heating regime consists of robust heating process control where temperature is the control parameter. Even if the convective heating process till semi-solid state requires a longer time, it allows the heating of several billets at the same time. However, given the long duration of heating, an undesired increase in grain occurs during reheating [19] and the mechanical stability of the billet decreases the more it is maintained in a semi-solid state [6]. The radial and axial cohesion forces of the semi-solid material in the volume of the billet decrease with the heating time and become less than the force of gravity. Under these conditions, the cylindrical billet in the furnace in vertical position collapses and takes the shape of a conoid.

Induction heating is a more commonly used method in semi-solid applications because it is clean, compact, and has a shorter heating time. In this case, the input electrical power is controlled. However, due to the several electromagnetic phenomena associated with the “skin effect”, the electromagnetic transverse edge effect and some others, the current distribution within the billet is not uniform, which causes temperature gradients [71,72]. As well, if either the size or the material of the billet, or the induction heater frequency is changed, the reheating parameters are affected. For large billets, induction heating is more difficult to implement. For billet diameters of 76–150 mm, induction heating furnaces in the medium frequency range are to be preferred [6]. In the case of semi-solid state billet heating, it is possible by inductive heating to have a more solid outer skin allowing the billet to be handled with a gripper to forming stage. On the other hand, an outer surface with a lower liquid fraction allows gripping with pliers and moving the semi-solid piece.

**Fig. 19:**
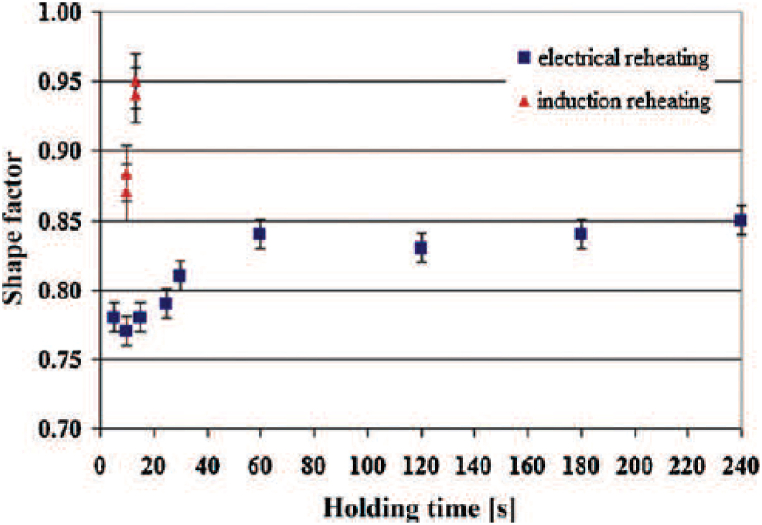
Shape factor vs. time [18].

Neag et al. [18] comparing the two heating systems (used to provide quasi-isothermal conditions for backward extrusion of semi-solid 7075 aluminium alloy), mentioned that heating the billet using an electric resistance furnace involves a long heating time and provides a grain shape factor (strongly dependent on holding time and reheating processes) inferior to that obtained by induction heating. In this context, Fig. 19 presents the relation between the variation of the shape factor and isothermal holding times. So, comparing the two heating systems, they notice two major differences:

i) using induction reheating gives more spherical grain shape (still faceted but without sharp corners);
ii) microstructure of globular solid grains surrounded by a thin liquid film uniformly distributed over the cross-sectional area; this homogeneity of structure is suitable for thixoforming.

The suitable conditions for the thixoforging process can be obtained by reheating the billet [6]:

• rapid in order to maintain the initial globular microstructure and avoid grain growth, as well as for economic reasons;
• accurate to obtain the required liquid fraction with the possibility of reproducing the conditions;
• homogeneous throughout the volume of the heated samples.

### 2.1 Microstructure/morphology characterisation

The microstructure evolution of reheated material to the semi-solid state is influenced by the phase fractions and the semi-solid alloys’ interval characterization, and their microstructure is necessary in order to know their thixoformability.

Over the years, many authors have confirmed that the feedstock material microstructure, characterized by grain morphology and grain size (usually defined as their mean diameter), results after reheating to the semi-solid range, influences the rheological behaviour and the final mechanical properties of the thixoformed parts. If the semi-solid material is prepared by reheating, it is important to know if the microstructure has reached the desired characteristics. Thus, it is necessary to know the correlation between the structure and the temperature, and thus the holding time with its apparent viscosity.

It is known that the liquid fraction, the degree of agglomeration, and the initial grain shape and size under different reheating conditions have an important effect on the rheological behaviour of semi-solid alloys (strong effect on viscosity) and on the mechanical properties after the semi-solid forming process by influencing the microstructure of the final part. Consequently, microstructure evolution control is necessary to ensure the reproducibility of the thixoforming process.

The shape factor is one of the rules used to evaluate the semisolid slurry because it strongly influences the material flow. The shape factor (F) is estimated in the literature using equation (1):

(1) $$F=4\pi A/P^{2}$$

where A represents the area of the globule and P its perimeter, respectively. A shape factor equal to 1 is the ideal case of perfect globular grains, and lower values correspond with an increasing amount of irregularity.

Wang et al. [73] proposed an empirical equation (2) concerning the connection of the grain size in the as-cast microstructure and the size of the globulites after partial remelting to determine the isothermal holding time required to achieve a suitable semisolid state, described as:

(2) $$Remelted\;particle\;\text{roundness}=1+0.09\frac{{\text{grain}\;\text{size}}_{\text{as}‐\text{cast}}‐110}{\text{holding}\;\text{time}+0.5}$$

Seo and Kang [74] expressed the grain shape as roundness (*R*) with the relation (3), and grain size as equivalent diameter (D_eq_), or the diameter of a circle whose surface equals the grain area, with the relation (4):

(3) $$R=\frac{\sum_{N=1}^{N}C_{N}^{2}/4\pi A_{N}}{N}$$

(4) $$D_{\text{eq}}=\frac{\sum_{N=1}^{N}\sqrt{4A_{N}/\pi }}{N}$$

where *C*_*N*_ is the circumference of a grain, *A*_*N*_ is the area of a grain, and *N* is the number of grains. The roundness equal to 1 represents the perfect case, when all grains are circular.

During reheating, when material spheroidization occurs, liquid pockets are entrapped in the primary globule and cannot contribute as a lubricant to the final semi-solid forming step, [6]. A globular grain shape can achieve a smooth motion in the liquid phase during the forming process, avoiding grain agglomeration. If the grain shape is not globular, agglomeration occurs due to insufficient lubrication. Ito et al. [75] noted that the solid particles can agglomerate even for moderate values of solid fractions. Atkinson [3] mentioned that in semi-solid metallic systems, agglomeration occurs because particles are colliding and, if favourably oriented (to form a low energy boundary), form a boundary. The agglomerated particles sinter and form a connected skeleton, while the liquid phase may be entrapped in the solid phase or spatially continuous and free to flow [1]. A low entrapped liquid in the solid phase and small, round alpha particles lead to decrease the stress needed to initiate the flow [76]. Quantitatively, the entrapped liquid can be estimated by 2D microstructure image analysis of polished areas, but 3D analysis allows a more detailed investigation and obtains more reliable data, as in the case of the shape factor and globule size.

**Fig. 20:**
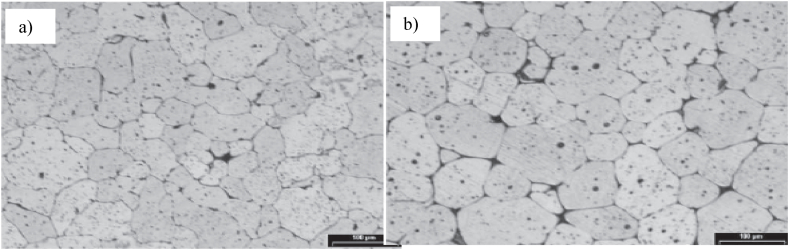
Micrographs of 7075 aluminium alloy isothermal reheated at 580 °C; holding time: a) 30 min; b) 240 min, [18].

**Fig. 21:**
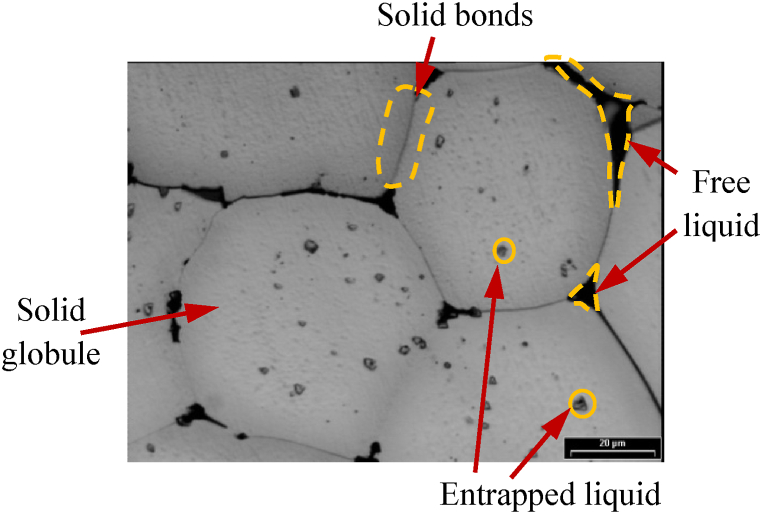
Microstructure required for thixotropic behaviour (f_S_ = 0.82), [18].

Neag et al. [18] observed an almost perfectly globular recrystallized microstructure with the average grain size <70 μm (see Fig. 20 a), rapidly provided after water quenching (to mimic the thermal treatment before thixoforming), with intragranular liquid droplets homogeneously distributed in the spherical solid particles and particles surrounded by solid bonds and the not entrapped liquid/free liquid (see Fig. 21). Prolonging the holding time leads to an increase in the mean grain size up to 75 μm (see Fig. 20 b), while reducing the grain size dispersion. Bolouri et al. [48] also observed that prolonging the holding time leads to an increase in the number of intergranular fluid droplets but also to an increase in their average size (coalescence and Ostwald maturation).

Jiang et al. [51] report that, when the isothermal temperature was increased to 615 °C, they observed a decrease in the amount of intragranular droplets that moved to the grain boundary and kept interconnected with the liquid phase on the grain boundary. They concluded that grains coalescence and Ostwald ripening occurred together in coarsening of grains. They also observed that the RAP microstructure gives a higher coarsening rate at 590 °C in comparison to the SIMA microstructure. Jiang et al. [51] studying the two frequently used preparation routes of 7075 alloy for semi-solid state processing, observed that the RAP microstructure provides a greater coarsening rate at 590 °C than the SIMA microstructure (329 m^3^ s^−1^ versus 316 m^3^ s^−1^).

**Fig. 22:**
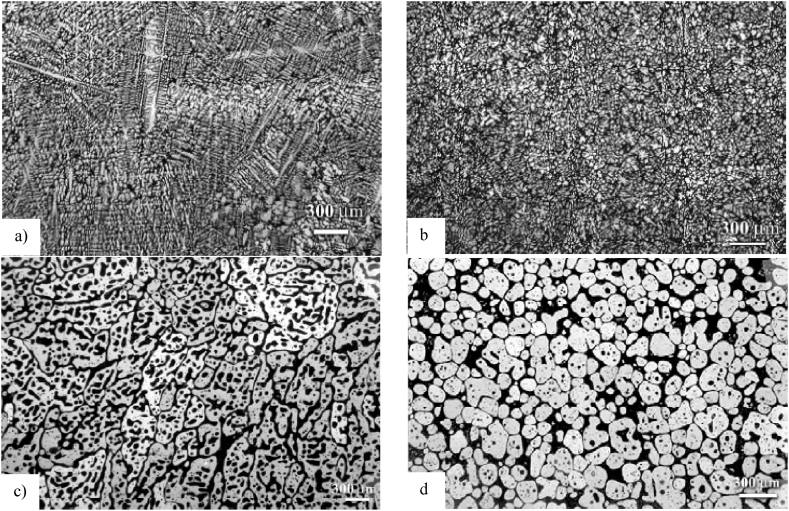
As-cast microstructures: (a) coarse structure from high-temperature-pouring (725 °C); b) fine-grained structures of dendritic; Microstructural evolution during partial remelting of c) coarse-grained structure; d) fine-grained dendritic structure. Samples were isothermally held at 580 °C for 30 min [66].

Wang et al. [66] for instance, reported that A356 aluminium alloy with dendritic grains with a diameter larger than 800 μm is not able to evolve into a globular structure during partial remelting and isothermal holding (Fig. 22 (a) and (c)). They observed that dendritic grains with a size between 200 and 600 μm after a long isothermal holding, evolve to a globular structure with relatively large particle sizes (Fig. 22 (b) and (d)). While grain size less than 200 μm after a short isothermal holding time evolves towards a spherical globular structure. Cast alloy with grain size of 110 μm or less doesn't require isothermal holding time to evolve to a spherical globular structure.

Chen et al. [77] obtained fine spheroidal microstructures with grain size of 40 μm without unrecrystallised grains by the hyperthermally and subsequent isothermally reheating regime. They suggested that the rapid heating rate and increased superheat degree have the advantages of forming more liquid to dissolve pinning particles, a higher nucleation rate of recrystallization, and a lower coarsening rate by reducing the coalescence of grains.

On forming, the size of the spheroid can limit the capacity of the flow in thin sections of the die walls. For a metallic alloy, a globulitic solid phase with grain sizes of <100 μm is favourable for thixoforming, [6]. In the case of thin component areas, to attain a correct filling, the smallest component widths to be filed without causing noteworthy separations of solid and liquid phase components should not be less than 20–30 times the grain radius [4]. In addition, Atkinson and Liu [78] noted that if the spheroid size upon forming is greater than about 100 μm, the final mechanical properties of the thixoformed component are likely to be unacceptable.

Kiuchi and Kopp [79] recognized that if the grain surface is not sufficiently globular, the grains stick together when sheared, and no thixotropic behaviour is possible. Kapranos et al. [80] studding 5182 aluminium alloy considered that a shape factor above 0.6 is appropriate for semi-solid forming. Bolouri et al. [28] reported significant effects on shape factor improvement by prolonging holding times and increasing temperature within the mushy zone. Neag et al. [18] reported a shape factor strongly dependent on holding time and reheating systems that increases up to a steady state value of 0.85 after 60 min of reheating in the resistance furnace, while it increases up to 0.95 after only 13 min of induction reheating for the same 7075 alloy billet dimension. Mohammadi et al. [52] obtained an equiaxed microstructure with a shape factor between 0.7 and 0.75 after a holding time less than 30 min, also on a 7075 alloy, at a solid fraction of about 80 % (lower solid fraction than in Neag et al. [18] study).

**Fig. 23:**
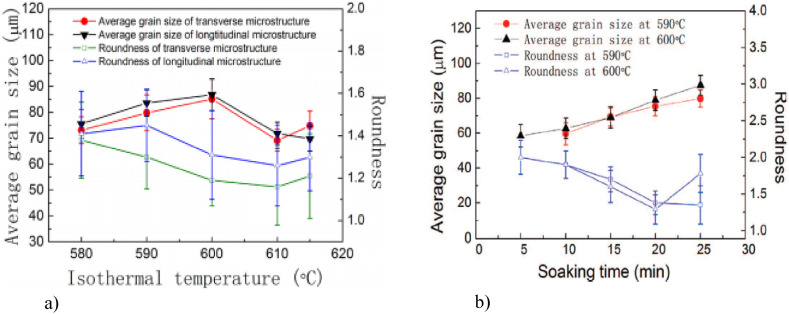
Average grain size and roundness of the microstructure of 7075 aluminium alloys fabricated by **RAP** a) soaked for 20 min at different isothermal temperatures and b) at 590 °C and 600 °C [51].

Jiang et al. [51] report roundness varies for 7075 aluminium alloys fabricated by RAP in a range of 1.2–1.4 for a different isothermal temperatures range of 580–600 °C and the grain size range in sizes from 70 μm to 87 μm, see Fig. 23(a). Moreover, they got a good spheroidization at soaking time of 20 min at 590 °C and 600 °C and an increase of grain size with increasing soaking time for 20 min at 590 °C and 600 °C, see Fig. 23(a and b). The grain size is in the same range of magnitude as the one reported by Neag et al. [18] and Mohammadi et al. [52] for similar conditions. As expected, a longer soaking time led to coarsening of microstructures.

Jiang et al. [51] also note that they obtained better spheroidization, larger spheroidal grains, and lower spheroidization temperature when preparing 7075 alloy for semi-solid processing by RAP method compared to SIMA. Kapranos et al. [73] investigated two different methods to obtain a cheap microstructure of semi-solid feedstock and demonstrated, by metallographic investigations, the superiority of the microstructure obtained by heating and isothermally maintaining 5182 aluminium alloy billets prepared by the rolling route over the casting route. Also, De Freitas et al. [86] observed that, even though in extruded 2024 alloy samples the texture is stronger than in rolled samples, after maintaining a longer time in the semi-solid state, the particle size growth rates for rolled samples and extruded material are essentially similar.

The effect of the microstructure of the feedstock material on the mechanical properties of the thixoformed part has been the subject of many studies published by previous researchers. At high solid fraction, Sheykh-Jaberi et al. [82] find that the B206 alloy has a higher yield strength than the A356 alloy, and there are a number of contributing factors (morphology, grain size, etc.).

The results obtained from these studies show that a decrease in both secondary dendrite arm spacing, and grain size improves tensile properties, especially at high solid fractions where hot tearing is most prevalent.

## 3 Conclusions

The thixoforging of metal alloys allows for the manufacture of parts to reduce the number of shaping steps, reduce the raw material involved in the manufacturing process, and as a result, have a more economical and ecological production cycle. For aluminium alloys, this process also increases the characteristics compared to the casting process.

In the case of aluminium alloys, to get the most out of this process, it must be mastered by:

• The material chosen must not only meet the needs of the product, but also the conditions of thixoforgeability, including a temperature range, solidus-liquidus, as wide as possible. Wrought aluminum grades are generally more suitable for thixoforging.
• The study of the temperature range, solidus-liquidus, of aluminium and can be done by:
✓ experimentally, DSC; requires means and does not correspond to the used heating conditions of a slug, due to the scale difference with the sample for a DSC and the speed heating difference,
✓ numerical simulation; good results, but the number of grades is limited;
✓ remarks: Attention should be paid to the rate of heating and cooling and to additives that can significantly influence the thixoforgeability temperature range. A high heating rate decreases the initial melting temperature.
• The preparation of the materials to generate a globular microstructure of the raw material is used to favour the flow of the alloy during shaping. This preliminary stage remains a costly one, and it is necessary to consider the possibility of using some of the aluminium in its raw state. For the time being, there are no significant studies on this last point in the case of thixoforged aluminium as for steel alloys.
• The heating of aluminium in the semi-solid state can be achieved by two heating modes, electric furnace and induction furnace; other systems are not described in the literature reviewed. The first mode allows a large number of parts to be heated simultaneously and in a quasi-isothermal condition before transfer to the press. However, the heating time of the first parts is long. The second mode is unitarily much faster; however, the temperature of the slug volume is not uniform, and there is a liquid fraction gradient. This last point may be an advantage for handling the slug if the solid fraction outside the slug is high.
• The nature of the microstructure of the aluminium can be a factor in improving its use in the thixoforging process; circularity and size are the most decisive. On the other hand, this microstructure conditioning has a cost and can be a limitation to the use of the process. A process model needs to be developed to integrate this step during reheating of the material to the semi-solid state.

## Data availability statement

Data included in article/supplementary material/referenced in article.

## CRediT authorship contribution statement

**Adriana Neag:** Writing – review & editing, Writing – original draft, Methodology, Investigation, Conceptualization. **Eric Becker:** Writing – review & editing, Methodology, Conceptualization.

## Declaration of competing interest

The authors declare that they have no known competing financial interests or personal relationships that could have appeared to influence the work reported in this paper.

## Figures and Tables

**Table A:** Summary table of the literature on aluminium alloys used in semi-solid processes (n.d. = not determined) for heating rates between 10 and 200 C°/min.

	Alloys	Authors	ΔT_max_ = T_L_-T_S_ [°C]
Wrought alloys	7075	[9,11,13,14,18,20,28,29,48,49,[51], [52], [53],^58^,^59^,^66^,^77^,^83^,[88], [89], [90], [91], [92]]	160
	2014	[9,11,47,59,88,93]	130
	6082	[6,9,88,94,95]	90
	6061	[84,85,96,97]	70
	7005	[15,69]	59
	7010	[11,88]	n.d.
	6063	[12]	23
	5013	[99]	n.d.
	5182	[80]	190
	2024	[11,56,84,86]	n.d.
Foundry alloys	390	[62,63,70]	157
	B206	[87]	140
	355	[11]	129
	201	[59,88,93]	122
	238	[11]	112
	308	[11]	107
	319	[6,11,13,33,98,100]	106
	380	[11,81,82,102]	89
	357	[16,47,62,67]	83
	356	[11,16,[54], [55], [56], [57],^60^,^64^,^68^,^73^,^74^,^84^,^87^,^98^,[101], [102], [103], [104], [105], [106], [107]]	80
